# Dielectric polarization-based separations in an ionic solution

**DOI:** 10.1039/d3ra03169a

**Published:** 2023-07-24

**Authors:** Gaurav Anand, Samira Safaripour, Craig Snoeyink

**Affiliations:** a Department of Mechanical and Aerospace Engineering, University at Buffalo Buffalo USA gauravan@buffalo.edu craigsno@buffalo.edu; b University at Buffalo 211 Bell Hall Buffalo 14260 NY USA

## Abstract

A novel non-electrophoretic, electric field-based separation mechanism capable of transporting ions based on their dielectric properties is presented here for the first time. Though this polarization-based mechanism behaves similarly to dielectrophoresis, the separation mechanism is remarkably very efficient at small length scales compared to any dielectrophoretic separation mechanism for particles. For an applied electric field of strength as low as ∼0.75 MV m^−1^ across a 100 μm channel, the working solute – sodium fluorescein – is shown to decrease in its concentration by ≈20% in electric field region relative to the non electric field region. The existing macroscopic theoretical models like electrohydrodynamics and equilibrium thermodynamics are shown to underestimate the concentration change by two orders of magnitude for the same electric field strength. This surprisingly large difference between theory and experimental results suggests that the electric field-based equilibrium thermodynamic model lacks a key physics.

## Abstract 2

A novel non-electrophoretic, electric field-based separation mechanism capable of transporting ions based on their dielectric properties is presented here for the first time.

## Graphical abstract

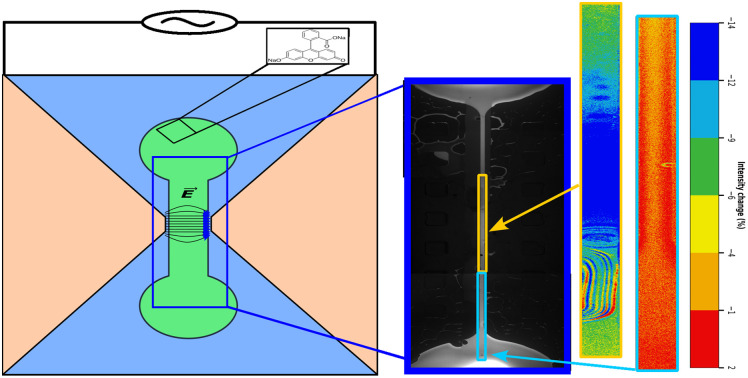

## 1 Introduction

Electric fields (E-field) are often used for the separation of ionic solutes through electrophoretic effects which transport the ions along E-field lines. Here we present for the first time a non-electrophoretic, polarization-based ionic separations method, which transports the ions along the gradient of E-field energy. This separations mechanism works to increase the permittivity of the solution within the regions of high E-field, typically transporting solutes to regions with lower E-field. The changes in the thermodynamic state resulting from the change in permittivity of the solution due to E-field induced polarization effects are not novel,^1,2^ the effects of E-field on equilibrium temperature and pressure have been studied extensively.^3–5^ To our knowledge though, there have been no experimental or modeling efforts on the effect of field-induced polarization on chemical separations in an ionic solution.

In contrast, electrophoretic-based chemical separation methods are common. Electrophoresis, as an example, utilizes the net charge on an ion and the strength of the E-field to push or pull it along the E-field lines.^6^ The transport rate is a function of electromobility and can be used to selectively remove species from a complex solution, for example, in a capillary.^7^ Other electrophoretic-based separation methods like electrodeionization and chronopotentiometry utilizes selective membranes, where ions are transported cross-stream towards electrodes fitted with membranes to retain them.^8–10^ Many other variations on these separation techniques have been developed to facilitate separations of ionic solutes from solution.^11–14^ Electrophoretic forces in combination with various Alternating Current (AC) E-field induced flows – AC electrosmosis and AC electrothermal – have also been conceptualized to concentrate ions at a particular location.^15–17^ Except for recent work by An and Minerick,^18^ to our knowledge, all reported field-based chemical separation methods rely on charge-transfer effects.

The separations mechanism presented here behaves qualitatively like the traditional dielectrophoretic transport of particles which are transported along the gradient of an E-field based on the relative dielectric properties of the particles and suspension fluid.^19–21^ Because it is not based on charge-transfer effects, both dielectrophoresis and the presented chemical separations method do not require electrical contact with the solution, permitting the use of blocked or insulated electrodes. The traditional dielectrophoretic transport based on Clausius-Mossotti function has been successful at modeling the transport of particles.^22,23^ However, since traditional dielectrophoretic transport scales with the cube of the particle radius, which in principle makes it difficult for molecular separations, it “massively underestimates” the transport rate in macromolecules of molecular weights in range of ∼1 kg mol^−1^ like proteins and DNA.^24^

The reason for failure of the traditional dielectrophoresis analysis method have roots in the limitations of this method to consider the microscopic changes in the molecular structure. To correct the limitations of traditional dielectrophoresis, various theories have been developed based on changes in molecular polarization while considering the microscopic structural changes.^25,26^ While these modified dielectrophoresis theories have come better at predicting the transport of molecules like proteins, no attempted theory has sufficed to accurately predict the dielectrophoretic transport for the entire range of solute sizes. As an alternative to dielectrophoresis based models, we propose a thermodynamic approach, the first step of which is presented here. The first objective of this study is to perform preliminary experiments to characterize a dielectrophoretic-based chemical separation method that can separate ionic solutes from a solution which has not been reported earlier. The second objective is to develop a preliminary thermodynamic model which qualitatively captures the behavior of the non-dielectrophoretic separation method.

In addition to the thermodynamic model, the experimental results presented here suggest, the dielectrophoretic-based chemical separations method is surprisingly effective for ionic solutes. Using a novel microfluidic system that can withstand the high E-field utilized in this study, we image the temporal and spatial variation in concentration of sodium fluorescein (NaFl) as it is transported and separated by the applied E-field. The resulting spatial variations in concentration are then compared to an equilibrium thermodynamic model utilizing the volumetric electrical energy, and the implications of the resulting discrepancies are discussed.

## 2 Material and methods

**Fig. 1:**
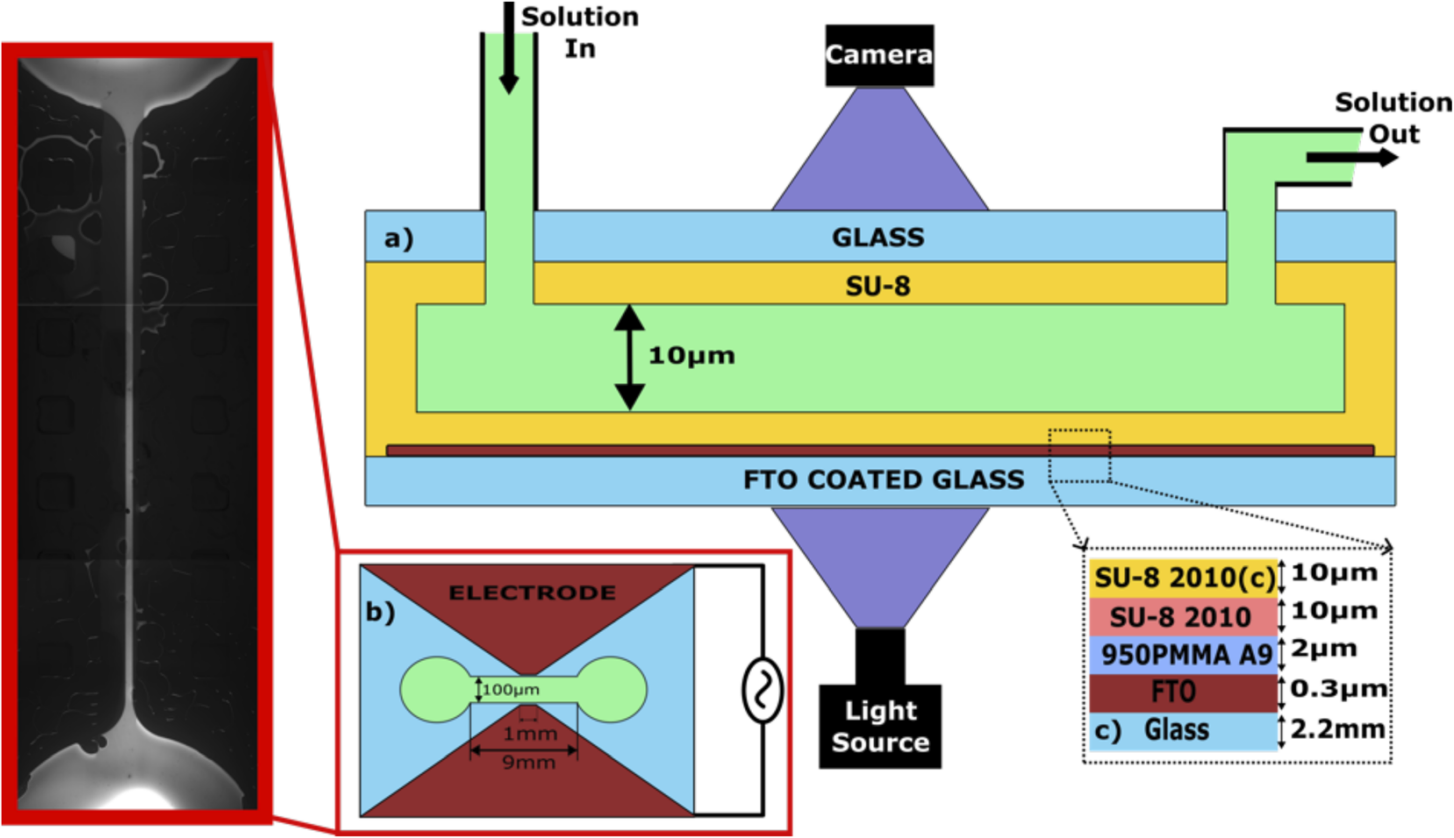
Schematic of the experimental setup. (a) Experimental setup with light and camera centered at the middle of the channel. The solution is pushed from the inlet at a volumetric flow rate of 180 μl h^−1^, and the depth of the channel is 10 μm. An FTO-coated glass slide is used as the bottom substrate and is coated with different layers of electrical insulation to form the dielectric layer, as shown in (c). SU-8 is used to form the channel and also to seal the channel after being coated on a microscope glass slide (Top glass slide). (b) shows the top view of the channel where the length, and width of the channel are 9 mm, 100 μm respectively. The electrode length which touches the edge of the channel is 1 mm. (d) An epifluorescent image of the channel with the sodium fluorescein solution flowing in the channel.

Fig. 1 shows the schematic of the experimental setup along with an epifluorescent image of the microfluidic chip and a schematic of the different layers of photoresists spin-coated to form the electrical insulation layer and to fabricate the channel. The dimensions of the channel are 9 mm × 100 μm × 10 μm along the length, width, and height respectively. An FTO (fluorine-doped tin oxide)-coated glass slide of size 1 inch × 1 inch with an electrode thickness of ∼320 nm and surface resistivity of ∼7 Ω cm^−2^ is used as the bottom substrate (supplied by MSE Supplies LLC).

To etch out the shape of electrodes on the FTO-coated glass as shown in Fig. 1(b), a simple masking and wet etching process was utilized. The substrate was first spin-coated with a positive photoresist (KL6005, supplied by KemLab Inc.) to form a 3 μm thick layer. The photoresist was then soft-baked at 105 °C for two minutes on a hotplate and then cooled down to room temperature. The baked photoresist was then exposed to UV light of wavelength 365 nm for 30 seconds while covering the part of the photoresist with a photomask of a shape similar to that shown in Fig. 1(b) designed on a polyester-based film (prepared by Fineline Imaging, Inc). The photoresist was developed with 0.26 N tetramethylammonium hydroxide (TMAH, supplied by LabPro Inc.) for 3 minutes to remove the exposed positive photoresist. Upon development, the substrate was covered with zinc oxide powder and 2 M hydrochloric acid (HCL) for 3 min to finish the etching process of FTO. The substrate was then cleaned thoroughly in an ultrasonic cleaner with acetone, ethanol, isopropanol (IPA), and hot deionized water subsequently for 15 minutes each.

The same cleaning procedure was followed for the top substrate, which was a microscope glass slide of size 1 inch × 1 inch with two drilled holes acting as an inlet and outlet for the channel. After cleaning the bottom substrate, four layers – two layers of polymethyl methacrylate (950 PMMA A9, supplied by Microchem Laboratory) of thickness 1 μm each and two layers of negative photoresist SU-8 (SU-8 2010, supplied by Microchem Laboratory) of thickness 10 μm each – of photoresist were spin-coated onto the etched FTO-slide. While the first three layers, *i.e.*, both layers of PMMA and first layer of SU-8, acted as electrical insulating layers to prevent any reactions at the electrode, the fourth layer, *i.e.*, the second SU-8 layer was spin-coated and patterned to form the channel as shown in Fig. 1. The rationale behind choosing PMMA and SU-8 as insulating layers is PMMA's excellent dielectric breakdown strength^27^ and SU-8's high chemical resistance.^28^

To spin coat each layer of PMMA, the same procedures as stated in the supplier's manual were followed.^29^ After the two layers of PMMA was coated, the first layer of SU-8 was spin-coated on top of it and uniformly exposed to UV light of wavelength 365 nm while following the same procedures from the supplier's manual.^30^ We coated another layer of SU-8 with the same procedures and exposed it to UV light using a film photomask with a laser-plotted channel. After the exposure, the unexposed SU-8 was removed by dipping the substrate in a SU-8 developer for 3 min and then washed with IPA. The substrate was then hard baked at 125 °C for 30 min.

To seal the channel, SU-8 was spin-coated on the top glass slide with the access holes covered with black tape. After soft baking the SU-8 layer, the top cleaned glass slide was brought in contact and aligned with the bottom slide. After aligning, the slides were placed under a hydraulic hot-press (manufactured by Dapress) set to 70 °C and the pressure was slowly increased to 120 psi and then maintained for 2 min. Since the temperature is above the SU-8's uncured glass transition temperature, the pressure ensures the uncured SU-8 will conform to the bottom channel.^31^ The channel was then allowed to cool down to room temperature while maintaining pressure. Finally, the entire chip is exposed to 365 nm UV light to permanently bond the two halves, followed by hard bake at 125 °C. To supply the power through the channel, electrical wires were connected to electrodes *via* a conductive, silver-based epoxy (supplied by Conductivex).

A voltage amplifier (supplied by Advanced Energy Inc.) that can supply a maximum voltage of 2000 Volts at 150 kHz was used to supply voltage across the channel. To maximize the voltage drop across an ionic solution, a square wave voltage with varying peak-to-peak amplitude (V_pp_) at a frequency of 20 kHz was supplied across the aqueous solution to reduce the steric effect within the double layer.^32^ When a 100 V_pp_ voltage is supplied across the electrodes with a 100 μm gap between them, an E-field strength of approximately 1 MV m^−1^ is created. The fluorescent ionic solution was prepared by dissolving Fluorescein sodium salt (supplied by Sigma Aldrich) in deionized water to achieve a final concentration of 0.5 mM with an electrical conductivity of ∼10 mS m^−1^. The solution was pushed through the channel *via* a syringe pump at a volumetric flow rate of 180 μl h^−1^, which results in an average velocity of 50 mm s^−1^ inside the 100 μm wide channel. This relatively high flow rate is several times higher than is currently possible using electrophoretic methods in a single channel.^33^

A thermodynamic model under the application of an external E-field developed earlier^32^ is used to predict the equilibrium concentration change for the fluorescein sodium (NaFl) dye solution confined in a channel. For such a system, *i.e.*, a closed system which is subjected to an external E-field, the Helmholtz free energy (d*F*′) is given by eqn (1),1
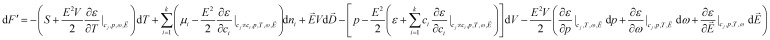
where, *S* represents entropy, *E* is E-field strength, *V* is volume, *ε* is permittivity of the solution, *T* is temperature, *c*_*i*_ is concentration of species *i* in molarity, *p* is pressure, *ω* is frequency, *D⃑* is electrical displacement vector, *μ*_*i*_ is chemical potential of species *i* in absence of *E⃑*, and *n*_*i*_ is number of moles of species *i*. In the presence of AC E-field of very high frequency, the term 
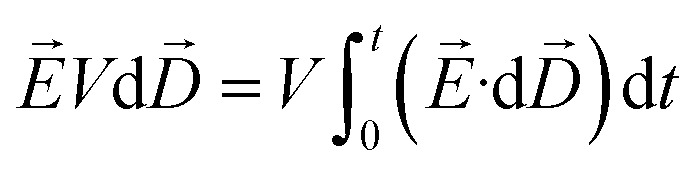
 is averaged to zero. Also, since the change in permittivity of the solution is negligible with change in frequency, pressure, and E-field strength,^34^eqn (1) reduces to the following:2
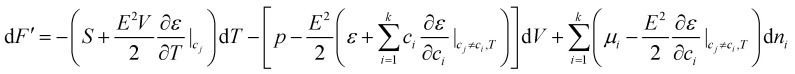


Joule heating, the increase in temperature due to the movement of ions under E-field, is also neglected here because the E-field strength required to see this effect is not high enough for the experimental solution conductivity.^35^ With the closed system assumption and conservation of moles, the difference in chemical potential of species *i* at equilibrium in E-field region (ER) and non E-field region (NER) is given by:3
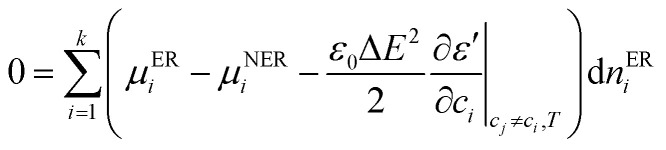
where *μ*^ER^_*i*_ and *μ*^NER^_*i*_ are the chemical potential of species *i* in ER and NER respectively, *ε*_0_ is the permittivity of vacuum, *ε*′ is the dielectric constant of the solution, Δ*E* is the difference in E-field strength between ER and NER, and *n*^NER^_*i*_ is the number of moles of species *i* in ER.

Assuming that the NaFl salt dissociates completely into sodium ions (Na^+^) and fluorescein ions (Fl^−^) in water and the usual definition for the chemical potential of species *i* – *μ*_±_ for ions, *μ*_*w*_ for water – are valid, the change in the number of moles of both ions and water can be predicted with eqn (4),4
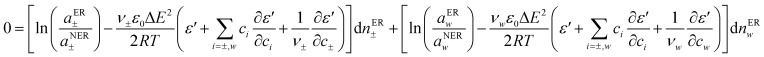
where, *a*^ER^_±_, *a*^NER^_±_ are mean molal activities of ions in ER and NER respectively, *ν*_±_, *ν*_*w*_ are the molar volume of ions and water respectively, *R* is the universal gas constant, *n*^ER^_±_, *n*^ER^_*w*_ are the number of moles of ions and water in ER respectively, and *a*^ER^_*w*_, *a*^NER^_*w*_ are mean molal activities of water in ER and NER respectively. Since the temperature and pressure of the system is fixed, a relationship between molar volume and the change in the number of moles of water and ions (*ν*_±_d*n*_±_ = −*ν*_*w*_d*n*_*w*_) is used to simplify the relationship between activities of species in eqn (4) to (5),5
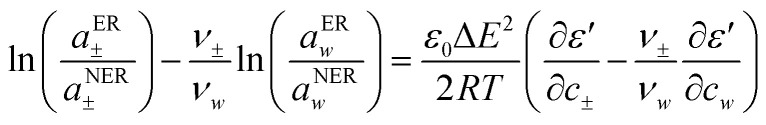


Assuming a dilute solution would mean that any change in waters' concentration and consequently activity can be neglected, resulting in final relationship for difference in number of moles in ions in ER and NER.6
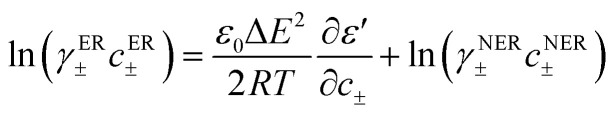


Though there are various methods to evaluate the activity coefficient of ions (*γ*_±_) in a binary solution,^36^ majority of the methods give approximately the same value for dilute solutions. In our case, we used Debye–Huckel extended law^37^ to evaluate the activity coefficient of Na^+^, Fl^−^ ions, where the parameter for effective diameter for ion was set to 3.^38^

## 3 Results and discussion

**Fig. 2:**
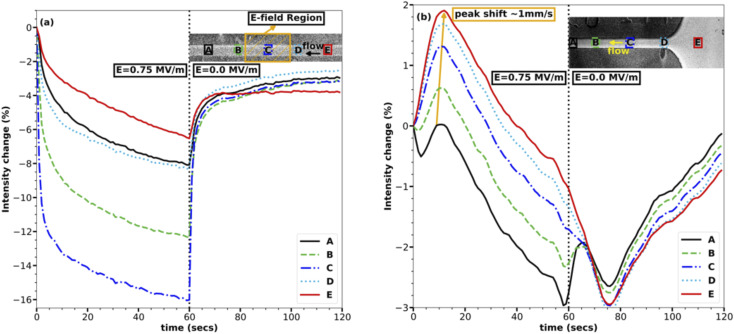
Change in fluorescence emission intensity of the 0.5 mM sodium fluorescent (NaFl) at the middle and the inlet of the channel. The insets represent the five different locations chosen in the channel, hatched area represents the electric field region, and the arrow shows the direction of the flow. (a) The line plots are changes in intensity averaged over a width of 100 px. (b) Line plots for change in intensity.

The concentration within the channel rapidly drops within and near the E-field region upon application of the E-field, as can be seen in Fig. 2(a). The analysis presented here is based on images taken at a rate of one frame per second. The E-field of strength 0.75 MV m^−1^ was turned on for one minute and then was turned off, as represented by two halves separated by a dotted line in each of the plots in Fig. 2. Fig. 2(a), which shows the middle of the channel, also has the electrodes at the center, represented with a yellow-colored hatched area. In Fig. 2(a), the instant dip in the intensity in the first half of the plot as soon as the E-field is turned on shows that the middle of the channel losses the maximum of the fluorescent in the first five seconds and following that initial drop the rate of decrease in intensity reduces to a point where the change is minimal at the end of the 60 seconds.

The rate of concentration decrease in response to the E-field is remarkable and suggests that the transport of the NaFl ions in/out of the middle of the channel is very fast as they must be transported upstream against the fast 50 mm s^−1^ average flow velocity within the channel. This transport rate is, to our knowledge, faster than any dielectrophoretic transport reported till now for ions.^23^ The rapid increase in concentration following the cessation of the E-field makes sense given the fast flow rate of the system: the fluid within the channel is completely changed within 0.2 s which is faster than the frame rate of our data. The decrease in the concentration of the ions in the E-field region also rules out the electrophoretic effect or any electrophoresis-based separation since electrophoresis pulls ions in the E-field region rather than losing them. The change in intensity due to the change in pH of the solution can also be ruled out because the electrodes were covered, and no reactions were observed anywhere in the channel. Also, as discussed above, the E-field strength and conductivity of the solution are not high enough to establish a temperature gradient in the channel due to Joule heating, which rules out the effect of the temperature on the emission intensity of NaFl. Though photo-bleaching of NaFl is possible over time, it was avoided by flowing the solution at a very high rate, ensuring fresh fluorescent dye for each exposure. Another advantage of the high velocity of the solution in the channel is, it also helps eliminate any unpredictable flow phenomena due to E-field.

In agreement with eqn (6), the regions with the highest E-field strength (*i.e.*, region C which is centered in the E-field region in Fig. 2(a)) undergoes the maximum change in intensity, reaching a concentration change of ∼16%. The effect of the E-field reduces as one moves away from the E-field region in either direction, rightly exhibited by the line plots for regions A, B, D, and E in Fig. 2(a), where the maximum intensity change reduces as the E-field strength reduces. Regions A and E are the farthest from the E-field region, and their corresponding line plots experience the smallest change in intensity of ∼5%. The non-symmetrical nature of the change in intensity about the E-field region, *i.e.*, regions A, B, and D, and E have different maximum intensity changes, is due to the advection of dye through the channel which is forcing solution from regions D, E to regions A, B, making regions D, E less depleted of ions.

The inlet of the channel reacts very similarly to the middle of the channel except for two very important and distinguishable characteristics as shown in Fig. 2(b). The first characteristic is the increase in intensity in all the regions upon application of the E-field except for region A, which is closest to the E-field region and experiences a loss in ions. The initial increase in intensity in these regions, *i.e.*, B, C, D, and E can be attributed to the transport of the ions upstream from the middle of the channel to the inlet and further downstream. The rapid loss of ions over the first five seconds from the middle of the channel, as shown in Fig. 2(a), coincides with the rapid gain of ions in the inlet as shown in Fig. 2(b), bolstering the hypothesis that the ions are being transported upstream from the E-field region to the non E-field region. The speed of this wave of increasing concentration was ∼1 mm s^−1^, when the position of the peaks was compared as depicted with an arrow in Fig. 2(b). This suggests that the transport of ions upstream is at least ∼1 mm s^−1^ faster than the average flow velocity of 50 mm s^−1^, further reinforcing the remarkable speed of this field-induced transport.

The second notable characteristic of the transport is shown in Fig. 2(b) is the gradual decrease in intensity everywhere in the channel approximately ten seconds after turning on the E-field. This feature can be attributed to the further transport of ions from the channel to the inlet and possibly up into the inlet tube after the first wave of high concentration originated in the E-field region passed through. Though it would be interesting to see the ions accumulating in the inlet tube, it has not been studied here due to the optical inaccessibility of this region. This does, however, suggest that the rate of transport is proportional to the concentration gradient. The initial transport rate is very fast because the E-field sets up a new equilibrium concentration change between the E-field region and the non E-field region. As the concentration difference asymptotically approaches this equilibrium condition, the rate of transport decreases.

Combining the results in Fig. 2 for both the inlet and the middle of the channel outlines the transport mechanism, *i.e.*, the first change in concentration is experienced in the E-field region which is rapid and then it spreads slowly in either direction pushing the ions further up or downstream. The very identical behaviour of the decrease in intensity with time in Fig. 2(a) to that of an exponential decay suggests that the concentration change is proportional to concentration or gradient of concentration at any time. Therefore, this transport mechanism is very similar to any diffusion based phenomena, except that the rate of diffusion is very high compared to any reported experimental or theoretical dielectrophoretic based transport at the same E-field strength.

**Fig. 3:**
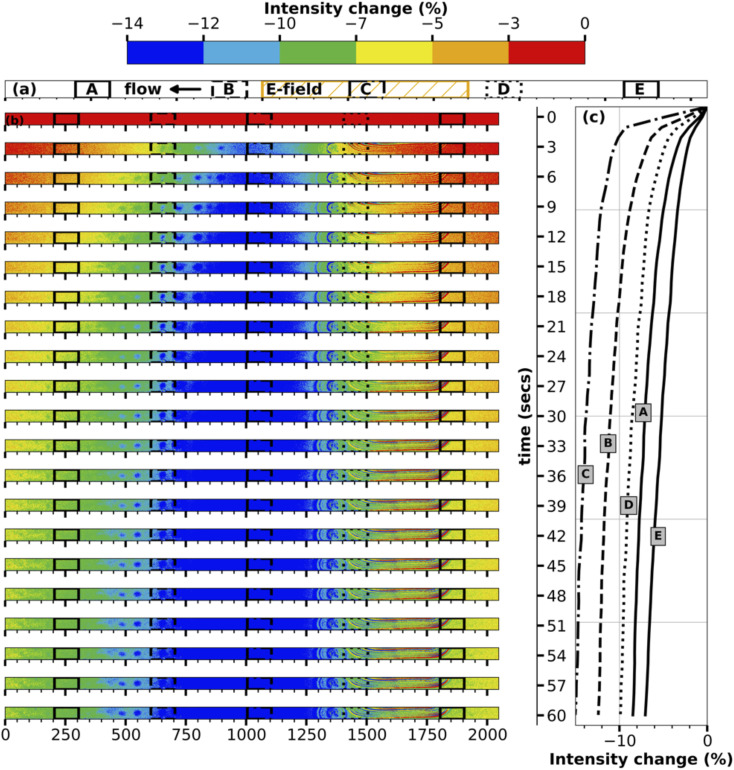
Time development of change in fluorescence emission intensity in the middle of the channel. (a) shows the locations in the channel chosen to show the intensity change in the line plots. (b) The contour plots are three seconds apart from the start of the electric field till the end of it. (c) are the line plots.

To better understand the transport mechanism, contour plots for intensity change are plotted for every three seconds between turning on the E-field and turning it off for both the middle and inlet of the channel, as shown in Fig. 3, 4 respectively. The contour plots for intensity change in Fig. 3 clearly show the initiation of transport of ions in the E-field region (region C, center of the channel), followed by progression of the transport of the ions in either direction into regions A, B, D, E. As time progresses, the E-field region experiences larger and faster depletion in ions, while the other regions experience a smaller and slower drop in concentration. The line plots for intensity change at five different regions accompanying the contour plots in Fig. 3 also show the same time progression of intensity change in the middle of the channel.

**Fig. 4:**
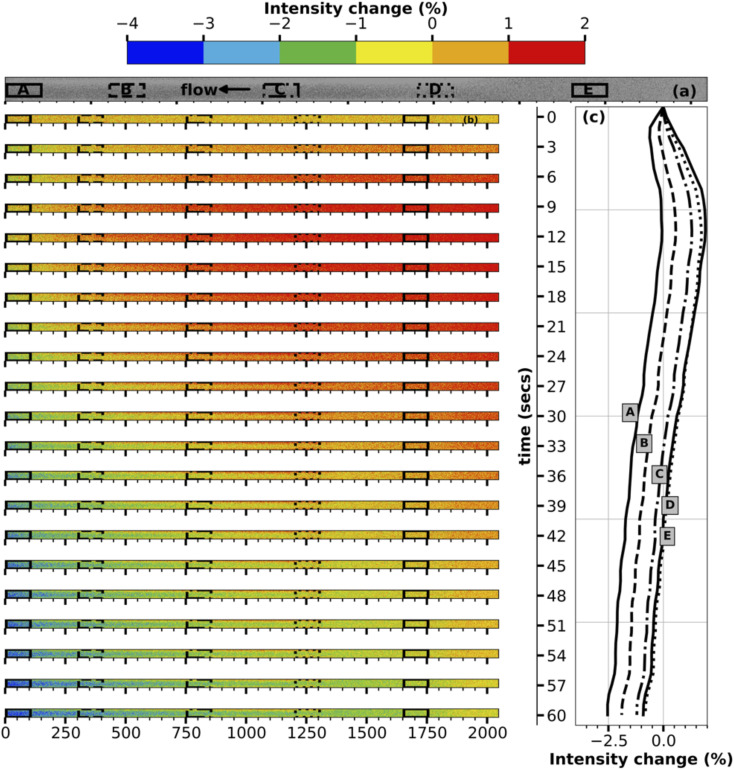
Time development of change in fluorescence emission intensity inlet of the channel. (a) shows the locations in the channel chosen to show the intensity change in the line plots. (b) The contour plots are three seconds apart from the start of the electric field till the end of it. (c) are the line plots.

The contour plots for the inlet of the channel shown in Fig. 4 are particularly fascinating as they showcase the transport of the high-concentration wave – colored red which represents an increase in intensity – originated at the middle of the channel then growing as it moves further downstream during the first ten seconds of the transport. Following the increase, the intensity starts to decrease everywhere in the channel as the ions continue to be transported up and downstream. While region A, being nearest to the E-field region, experiences it first, regions D and E being farthest from the E-field region receives the transported ions at last followed by loosing its ions such that concentration difference is negligible since the difference in E-filed strength between regions D and E is low. The line plots accompanying the contour plots in Fig. 4 support this hypothesis of the transport mechanism. At the end of one minute, the inlet of the channel experiences a maximum decrease in intensity of ∼3%, which is remarkable given the relative distance of the electrodes and the resulting low E-field strength.

While eqn (6) agrees qualitatively well with the experimental data, it under-predicts the equilibrium concentration for a given E-field strength by several orders of magnitude. The change in solution permittivity with dye concentration, ∂*ε*′/∂*c*_±_, was calculated by analyzing the current–voltage characteristics of this solution since a published concentration-based permittivity data is lacking for the NaFl solution. For the 0.5 mM NaFl solution, the change in permittivity with concentration was estimated to be ∼−10 mM^−1^. Though the value for the term ∂*ε*′/∂*c*_±_ can change with a change in concentration, it remains approximately constant in the dilute regime.^32^ At an E-field strength of 0.75 MV m^−1^, the change in NaFl concentration using the equilibrium thermodynamic model is ∼0.1%, two orders of magnitude lower than the experimental results presented here.

The discrepancy between the experimental results and the thermodynamic model can be attributed to two particular shortcomings of the proposed thermodynamic model. The first being the inability of the model to account for the microscopic structural changes resulting from the application of E-field on the solution confined between two electrodes.^39^ The change in permittivity with concentration in eqn (6) was measured at low electric-field strengths because rapid transport witnessed here varies the concentration making high electric-field permittivity measurements impossible. Structural changes induced by the high electric-fields may alter the change in permittivity with concentration in ways that are not accounted for in our model. The second possible reason for the discrepancy is the inability of the available activity coefficient models to include E-field effects. Activity coefficient models are experiment-based and therefore a good amount of data has to be collected before a model is developed. We propose that the development of models for E-field based activity coefficient and change in permittivity with concentration in presence of E-field are priorities for investigation.

## 4 Conclusions

The separations method presented here, while likely related to dielectrophoretic studies of macromolecules, is shown to be highly effective for a comparatively small fluorescent molecule, ionic FITC. The observed transport is remarkably fast and increases with the strength of the E-field magnitude as well as the difference with the equilibrium concentration condition. This suggests it is a dielectrophoretic molecular transport phenomenon, but an equilibrium thermodynamic model developed here under-predicts the equilibrium concentration difference by over two orders of magnitude, implying that there is physics not captured by the sole use of volumetric E-field energy. We hypothesize that the discrepancy is a result of field-induced entropic mechanisms not accounted for in the thermodynamic model, but the presented experimental results are not capable of testing this hypothesis.

The qualitative agreement of the thermodynamic model with our experimental results suggests that this transport mechanism could be broadly useful for microfluidics where the small length scales necessary to achieve the high electric fields are an advantage. For example, changing the size and shape of ions should increase the separation efficiency as it also have been observed in dielectrophoresis of high molecular weight solutes.^24^ Sensing ultra-low concentrated samples is another potential application. Since the presented separation mechanism is based on dielectric changes of the solvent due to addition another substance and E-field strength, one can believe that this mechanism will work efficiently even for ultra-low concentrated samples; all that is required is a change in permittivity with a change in concentration of the solute. The result is potential applications ranging from membrane-less water filtration to multiplexed bio-separation or concentration change owing to its ability to filter charged and uncharged species.

## Conflicts of interest

All authors declare that there is no conflicts of interest.

## Supplementary Material
